# Hell’s Itch: A Unique Reaction to UV Exposure

**DOI:** 10.2196/48669

**Published:** 2023-10-24

**Authors:** Katelin J Ball, Brandon W Muse, Bailey Cook, Alyssa P Quinn, Benjamin D Brooks

**Affiliations:** 1 College of Osteopathic Medicine Rocky Vista University Ivins, UT United States

**Keywords:** Hell’s Itch, social media, sunburn, sun, survey, skin, dermatology, dermatological, itch, itchiness, itchy, symptoms, experience, ultraviolet, UV, dermatologist, teledermatology, hair, nails, scratch

## Abstract

We present a survey-based exploration of Hell’s Itch, a severe dermatologic reaction often mistaken for sunburn, that reveals distinct symptoms including intense pain, unrelenting itching, paresthesia, and even suicidal ideation, differentiating it from a typical sunburn.

## Introduction

Hell’s Itch is an inciting dermatologic reaction that can occur after sun exposure and is often characterized by symptoms such as intense pain, itching, paresthesia, and suicidal ideation. A patient with Hell’s Itch described their experience as follows: “Dozens of white-hot sewing needles being repeatedly stabbed into my upper back. The pain is unrelenting and unbearable... It drives you completely insane.” Patient accounts like this distinguish Hell’s Itch from a typical sunburn.

One in 3 people in the United States experience a sunburn annually [1]. Sunburn is characterized by cutaneous erythema appearing 24 to 72 hours following sun exposure. The cause of sunburn is excessive exposure to UV radiation, commonly causing acute, transient inflammation in the skin [2]. Symptoms depend on sunburn severity and may include blistering, peeling, and pruritus due to peeling [2]. Like sunburn, Hell’s Itch is a manifestation of cutaneous damage after unprotected UV exposure. However, Hell’s Itch exhibits key differences in presentation. Hell’s Itch is reported as an acute, uncontrollable itch that causes stabbing pain when scratched [3]. In addition, Hell’s Itch results from a key inciting event such as topical cream application or water exposure.

Hell’s Itch is commonly misdiagnosed as sunburn. Misdiagnosis leads patients to seek medical advice from alternative sources such as social media. There are social media platforms with groups comprising up to 4000 members who share experiences and anecdotal treatment remedies.

Here, we explore the differences in symptomatology between Hell’s Itch and a typical sunburn using a patient survey.

## Methods

Hell’s Itch is not well characterized in the scientific literature, with only 3 published case reports to date [3-5]. Without objective diagnostic criteria available to establish a diagnosis of Hell’s Itch, our survey relied on subjective, self-reported data as the source to identify the distinguishing symptoms. We surveyed patients in social media groups with approximately 5500 total members to identify common symptoms, the incidence of symptoms, and symptom severity rated from 0 (none) to 10 (worst imaginable). Study inclusion criteria included adults aged ≥18 years with at least 1 episode of Hell’s Itch.

## Results

A total of 100 people with self-diagnosed Hell’s Itch completed the survey. The defining differences between the symptoms of Hell’s Itch and sunburn identified in the survey included unrelenting pruritus, intense pain, difficulty sleeping, paresthesia, and suicidal ideation (Table 1). Hell’s Itch symptoms commonly occurred after sun exposure and were often triggered by inciting events like water exposure (eg, showering, bathing, swimming, humidity, or sweating) or application of a topical cream (88/100, 88%).

**Table 1:** Hell’s Itch survey results (N=100).

Symptom	Prevalence (%)	Average symptom severity rating^a^
Pruritis	98	9.17
Pain	90	8.42
Difficulty sleeping	87	8.62
Paresthesia or tingling	77	7.61
Suicidal ideation	44	6.93
Peeling skin	33	6.20
Numbness	13	5.13
Other	32	8.94

^a^Symptom severity was rated from 0 (none) to 10 (worst imaginable).

## Discussion

The incidence rate of Hell’s Itch is currently unknown as it is commonly misdiagnosed as a sunburn. Our survey characterizes the prevalence and severity of key symptoms differentiating Hell’s Itch from a typical sunburn—intense pain, itching, paresthesia, and even suicidal ideation—which are distinctive symptoms absent with sunburn. However, the survey may be limited in its strength because it relied on patients self-diagnosing their condition. Future efforts should move beyond convenience samples and include studies to characterize pathophysiology, risk factors, and effective treatments.
